# Primary hepatoid adenocarcinoma of the lung with extremely elevated serum AFP: a case report and literature review

**DOI:** 10.3389/fonc.2024.1448219

**Published:** 2024-10-03

**Authors:** Chengsen Cai, Ningxin Zhang, Min Wang, Lianzhong Wang, Haisu Zhao, Xiaoye Zhang, Bin Li, Jun Wang

**Affiliations:** ^1^ Department of Respiratory and Critical Care Medicine, Second Affiliated Hospital of Shandong University of Traditional Chinese Medicine, Jinan, Shandong, China; ^2^ Department of Emergency, Second Affiliated Hospital of Shandong University of Traditional Chinese Medicine, Jinan, Shandong, China; ^3^ Department of Pathology, Second Affiliated Hospital of Shandong University of Traditional Chinese Medicine, Jinan, Shandong, China; ^4^ Department of Radiology, Second Affiliated Hospital of Shandong University of Traditional Chinese Medicine, Jinan, Shandong, China

**Keywords:** AFP, primary hepatoid adenocarcinoma, lung biopsy, immunohistochemical, chemotherapy

## Abstract

Primary hepatoid adenocarcinoma of the lung (HAL) is an exceptionally rare subtype of lung cancer that mimics the morphology and biological behavior of hepatocellular carcinoma. Although reports in the literature are limited, HAL is known for its high malignancy and poor prognosis, thus drawing increasing attention. We present the case of a patient with a mass-like consolidation with central necrosis initially misdiagnosed as inflammation at another medical institution despite a percutaneous lung biopsy. After ineffective anti-inflammatory treatment, she was referred to our hospital. We performed another lung biopsy, obtaining five samples from different angles, and eventually diagnosed her with HAL. Surprisingly, her serum alpha-fetoprotein (AFP) levels were extraordinarily high, leading to the successful diagnosis of HAL. Here, we present a case report and a related literature review.

## Introduction

Primary hepatoid adenocarcinoma of the lung (HAL) is a very rare subtype of lung cancer. As a rare disease, its diagnosis and treatment pose significant challenges. We report on a HAL patient who was initially treated at another hospital for a mass-like consolidation with central necrosis, with a computed tomography (CT)-guided lung biopsy suggesting inflammation. Fortunately, we diagnosed her with HAL; however, due to her advanced age and financial constraints, she only received one round of chemotherapy and passed away 4 weeks later.

## Case report

**Figure 1:**
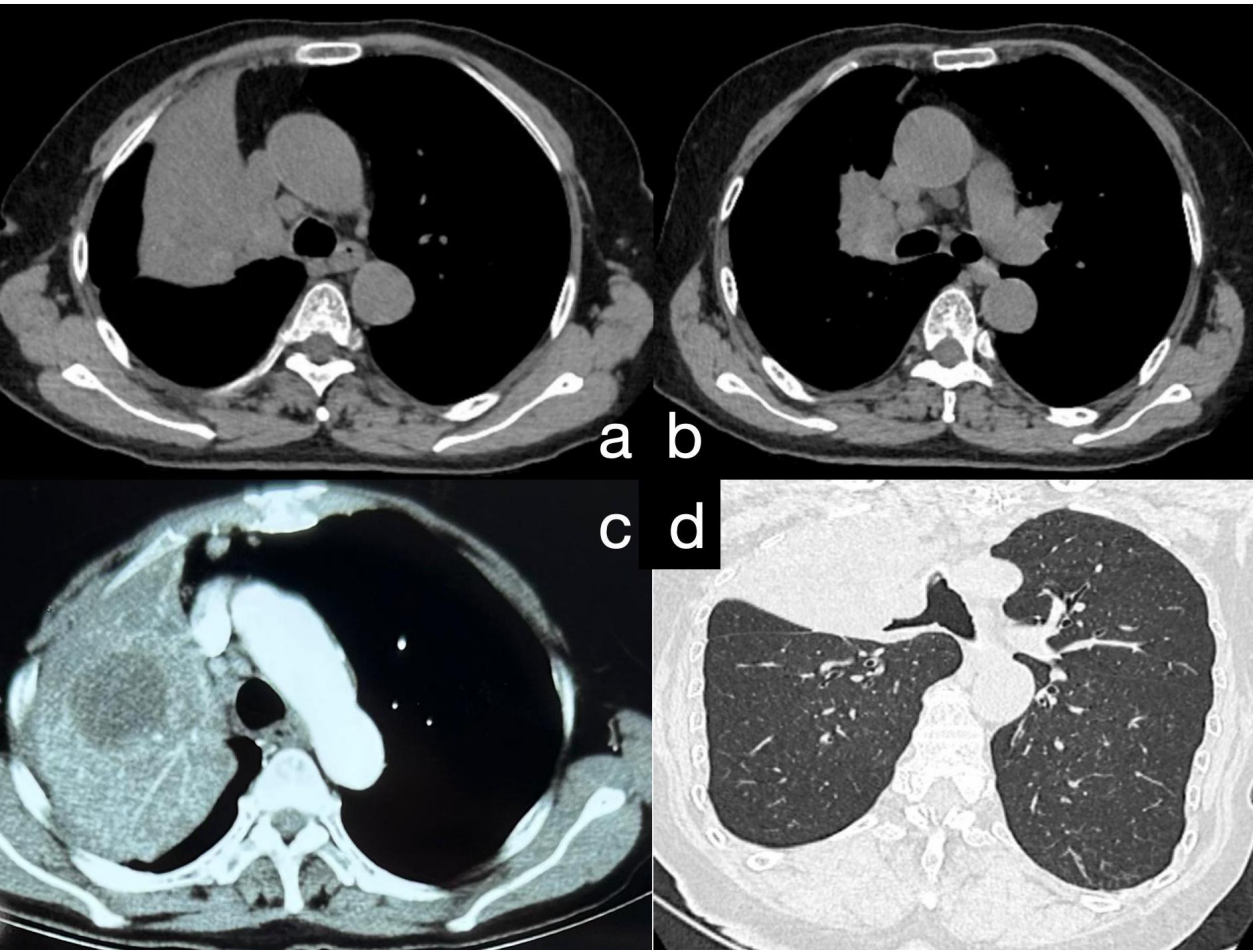
**(A)** Chest CT reveals a large area of consolidation in the upper lobe of the right lung. **(B)** Consolidation in the upper lobe of the right lung accompanied by multiple enlarged mediastinal lymph nodes. **(C)** Postcontrast chest CT demonstrates moderate enhancement in the consolidated area of the upper lobe of the right lung, with quasi-round, slightly hypodense lesions within the focus; the boundaries are still clear. **(D)** Following 3D reconstruction of the chest CT, bronchial narrowing and obstruction in the upper lobe of the right lung are evident.

A 75-year-old female patient was admitted to our hospital with recurrent fever, cough, and sputum production for over 8 months. The patient had a 30-pack-year smoking history and had quit smoking 6 months prior. She denied a history of hypertension, coronary artery disease, diabetes, or other chronic illnesses, as well as any abnormal personal, familial, or other psychosocial medical history. She underwent a CT-guided percutaneous lung biopsy at another hospital 1 month ago, with pathology suggesting inflammation (**Supplementary Material S1**). She underwent 1 week of anti-inflammatory treatment, but the effects were not significant; her symptoms of cough, expectoration, and fever persisted. When we admitted her, a physical examination revealed no signs of jaundice, bleeding, or rash on either the skin or mucous membranes. The superficial lymph nodes were not palpable or enlarged. Chest wall symmetry was maintained, with normal respiratory excursions and rhythmic breathing patterns. Auscultation revealed moist rales in the right upper lung field and slightly coarse breath sounds in the right lower and left lung fields, with no pleural friction rubs detected. No evidence of digital clubbing was found, and no peripheral edema was observed. An enhanced chest CT scan showed a large consolidation shadow in the right upper lobe, with a central ring of low density (**Figure 1**). Subsequently, the patient’s sputum was subjected to microbiological culture, which yielded negative results.

Surprisingly, laboratory tests revealed an AFP level of > 2,000 ng/ml (**Supplementary Material S2**), beyond our machine’s detection limit. Reassessment with another machine revealed an AFP level of 15,290.36 ng/ml (**Supplementary Material S3**) and a Carcinoembryonic Antigen (CEA) level of 28.1 ng/ml. In the differential diagnosis for such cases, in addition to a lung abscess, common pulmonary tumors such as lung adenocarcinoma, squamous cell carcinoma, and small cell lung cancer should be considered. This also includes other, less common types of lung cancer, such as sarcomatoid carcinoma. Moreover, teratomas should be included in the differential diagnosis list because some rare mature cystic teratomas can present on chest CT as lesions showing simple fat- or fluid-like densities (1). Importantly, studies have found that about 50% of patients with immature teratomas have abnormal serum AFP levels, with extremely high levels being observed in some cases (2). It should be noted that the imaging characteristics of HAL are not highly specific, and it is often challenging to differentiate based on imaging features alone; therefore, a comprehensive analysis combining other indicators is typically required. Based on the imaging findings and AFP results, we also considered teratoma in our differential diagnosis. However, the patient’s human chorionic gonadotropin (hCG) and estradiol results were normal, thereby ruling out a diagnosis of teratoma. Subsequent abdominal, pelvic, and cranial CT scans showed no significant abnormalities, ruling out tumors of hepatic, ovarian, or other origins. Thus, a disease of lung origin related to AFP was left as the diagnosis. Additionally, the patient’s slightly elevated white blood cell count, normal procalcitonin levels, and negative sputum microbiological culture did not support the diagnosis of a lung abscess.

**Figure 2:**
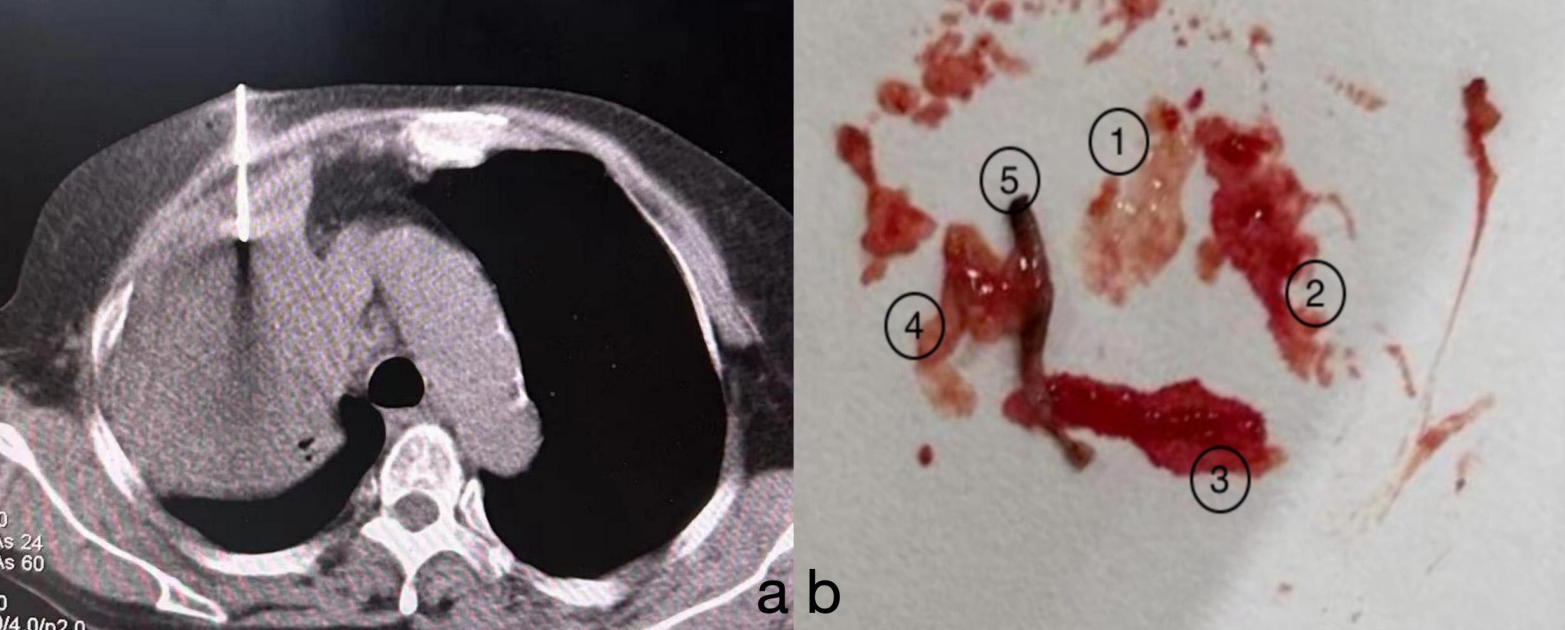
**(A)** After confirming the puncture site, a biopsy was performed on the lesion under CT guidance from central, anterior, posterior, left, and right angles. **(B)** A total of five biopsy samples were obtained. Samples 1 to 4 appeared as “fish-flesh-like” tissue, suggestive of necrosis. The fifth sample appeared greyish-white and was considered to be solid tissue from the lesion.

**Figure 3:**
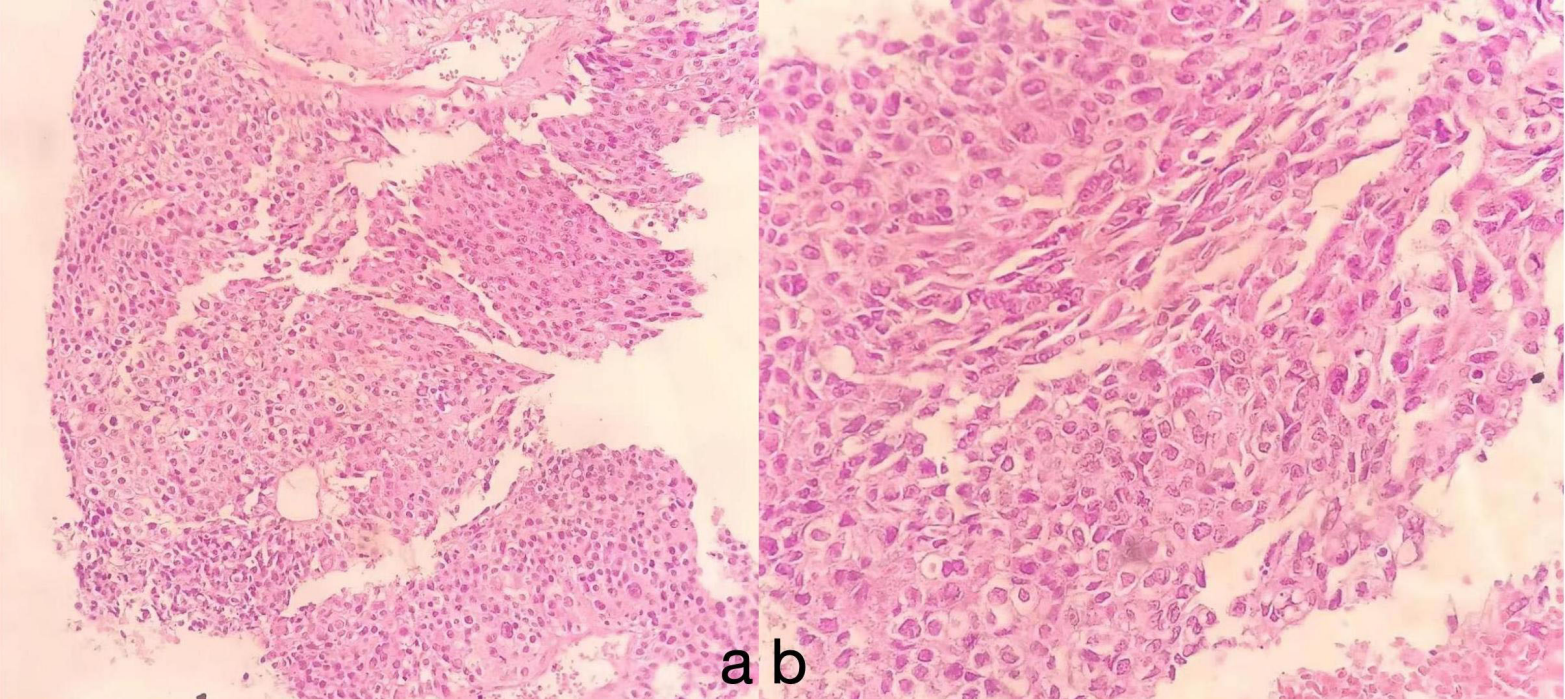
The tumor area exhibits hepatocyte morphology, with cells that are oval or polygonal in shape. The cytoplasm appears eosinophilic, and the nuclei are large and irregular, with an uneven distribution of chromatin. Mitotic figures are uncommon, but multinucleated cells and pleomorphic cells can sometimes be observed. Immunohistochemical analysis reveals features consistent with those of hepatocellular adenoma (HAL): **(A)** HE, × 10; **(B)** HE, × 40.

Finally, we decided to perform another lung biopsy (**Figure 2**), obtaining five samples from different angles, with four samples appearing necrotic. Lesion specimens were sent for pathological examination (**Figure 3**).

The immunohistochemical findings were as follows: Thyroid transcription factor-1 (TTF-1) (−), Napsin A (−), P40 (−), Syn (−), AFP (+), GPC-3 (+), Arg-1 (+), Hep-1 (+), CK (+), and CK7 (−). Based on these results and other examinations, a final diagnosis of primary HAL (stage IIIC, T4N3M0) was made.

**Figure 4:**
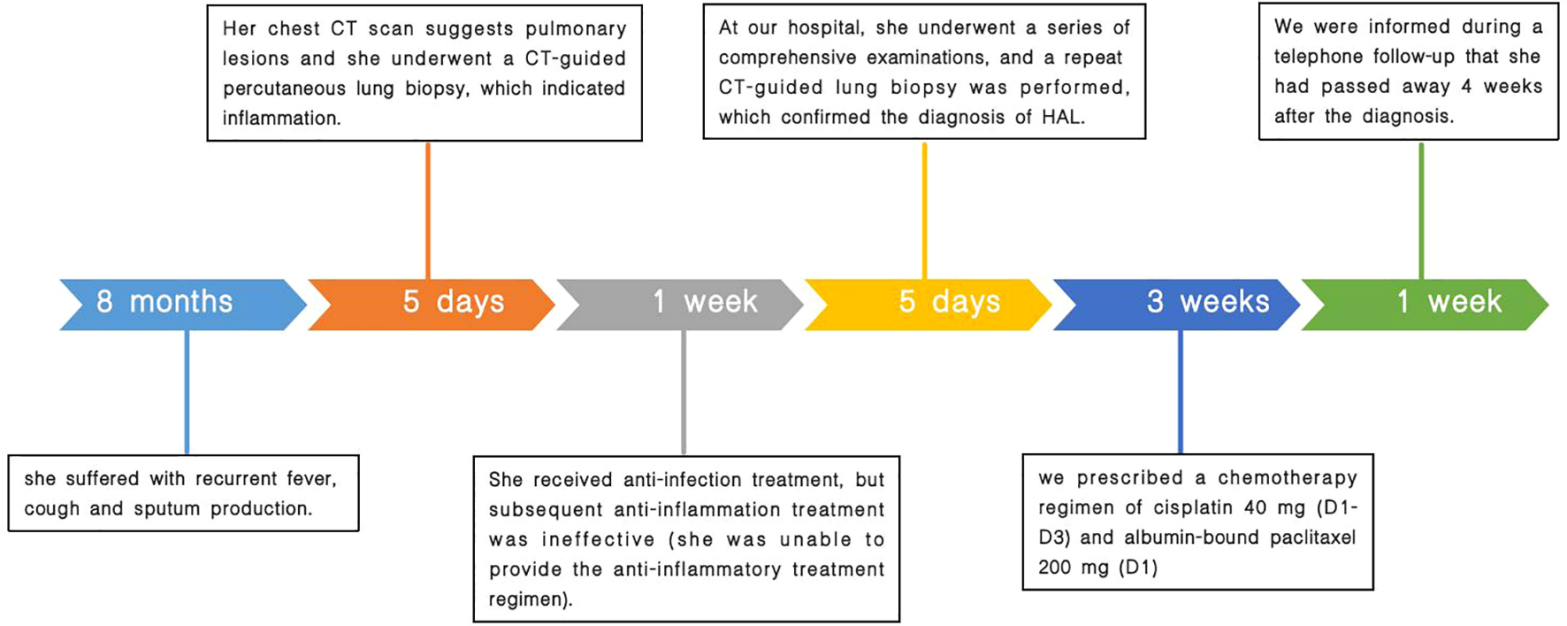
Timeline of the patient’s diagnostic and treatment process.

Based on the current lack of comprehensive treatment guidelines and our literature review, we considered the patient to have missed the optimal timing for surgery. Therefore, we recommended genetic testing of the lesion to evaluate the possibility of targeted drug therapy. We also offered radiation therapy and immunotherapy options. However, the patient could not afford genetic testing and immunotherapy and rejected the offered radiation therapy plan. Finally, according to the literature review (3, 4), we prescribed a chemotherapy regimen of cisplatin at 40 mg (D1–D3) and albumin-bound paclitaxel at 200 mg (D1), informing the patient that chemotherapy alone may lead to less favorable treatment outcomes. Nevertheless, due to the patient’s poor compliance, she did not undergo the standard serological and radiological examinations after treatment. Upon follow-up, we discovered that she passed away 4 weeks after diagnosis. A comprehensive timeline of the patient’s diagnostic and treatment process is presented in **Figure 4**.

## Discussion

Hepatoid adenocarcinoma (HAC) is a type of malignant adenocarcinoma resembling hepatocellular carcinoma that occurs in extrahepatic organs or tissues. Metzgeroth et al. reviewed 261 cases of HAC and found that the predominant locations were the stomach (63%), ovaries (10%), lungs (5%), gallbladder (4%), pancreas (4%), and uterus (4%). The median age of the studied patients was 65 years, with a range from 21 to 88 years. Common clinical manifestations included fatigue, weight loss, abdominal masses, and pain (5).

Ishikura et al. first proposed the occurrence of HAC in the lungs (termed HAL) in 1990 (6). The etiology of HAL remains unclear; it potentially arises from differentiation abnormalities during embryonic development, causing certain adenocarcinomas in organs like the lung to differentiate toward hepatocytes (7). HAL is highly malignant; Lei et al. analyzed HAL cases from 1975 to 2016, finding a median survival of 5 months (8). A history of smoking is a risk factor, correlating with a worse prognosis in HAL patients (9). Our patient had a long history of smoking and advanced disease at diagnosis and received only chemotherapy, resulting in a short survival.

The imaging features of HAL are nonspecific and lack typical characteristics. Most lesions are large (3.5–11 cm) and present primarily in the upper lobes, near the pleura or mediastinal pleura, often adjacent to the chest wall or major blood vessels (10). Enhanced CT typically shows necrotic changes (11), which is easily confused with pulmonary tuberculosis (12). Our patient underwent a CT-guided percutaneous lung biopsy at another hospital, which was suggestive of inflammation, although subsequent special stains for inflammation were negative. We noted a low-density area within the lesion center, possibly indicative of necrosis, which could lead to noninfective inflammation consistent with the characteristics of HAL. We speculate that the tissue obtained during her first biopsy might have originated from this necrotic zone, resulting in a false-negative outcome. However, serum marker testing or immunohistochemistry of lesion tissue can aid differentiation. Our patient’s CT showed a large lesion (10.7 cm) in the right upper lobe with central necrosis, typical of HAL. In the initial biopsy, necrotic tissue was likely obtained, causing a misdiagnosis, while solid tumor tissue was successfully obtained in the second biopsy.

The diagnosis of HAL relies on pathology and immunohistochemistry. Histologically, HAL resembles hepatocellular carcinoma, with large polygonal cells, abundant eosinophilic cytoplasm, and prominent nucleoli. Typical immunohistochemistry shows positivity for AFP, HepPar-1, and Glypican-3 (13). Our patient’s lesion tissue was positive for AFP, HepPar-1, and Glypican-3, which are crucial for diagnosis.

Additionally, the patient’s serum AFP level was significantly elevated. According to the literature, it is rare for HAL patients to present with such high levels of AFP. The present finding might be due to the overexpression of AFP in the hepatic-like differentiation areas of HAL (14). AFP can also test negative, with AFP-negative patients having longer overall survival (OS) (15). TTF-1 is often positive, indicating hepatoid differentiation and lung adenocarcinoma characteristics, although it is not essential for diagnosis. TTF-1 negativity is associated with poor prognosis in lung cancer patients (16).

Currently, there are no standard treatment guidelines or expert consensus for HAL, causing reliance mainly on case reports and case series. Research indicates that the treatment and management of HAL often follow guidelines recommended for non-small cell lung cancer (NSCLC) (19). Our literature review suggests that early-stage HAL treatment typically involves surgical resection, with an OS of up to 7 years, but this finding is based on treatments for stages I–II disease (8). Surgical resection is not the preferred modality for stages III–IV HAL patients, and no reports of a good prognosis following surgery at these stages have been published. The available treatments for advanced HAL include radiotherapy, chemotherapy, targeted therapy, and immunotherapy. For stage IIIA patients, concurrent chemoradiotherapy can achieve a better OS. Che et al. diagnosed a 48-year-old man who presented with an AFP level of 6,283 ng/ml with stage IIIA HAL. He was treated with radiochemotherapy, receiving 60 Gy in 2 Gy fractions over 30 sessions through intensity-modulated radiation therapy using 6-MV X-rays. This was concomitant with five cycles of paclitaxel and cisplatin. Following disease progression, the treatment regimen was switched to docetaxel and nedaplatin, resulting in an OS of 19 months (17). Cases of stage IIIC HAL patients undergoing only radiotherapy (50 Gy/25 fractions and 60 Gy/30 fractions) and achieving an OS of 12 months have also been reported (18). Chemotherapy often follows NSCLC protocols, with many reports of first-line chemotherapy with paclitaxel combined with platinum-based drugs, although the prognosis remains poor (3, 4). For stages III–IV patients, genetic testing of the lesion can be performed; if viable treatment targets and/or positive immunological expression are identified, targeted and/or immunotherapy may be considered. Targeted therapy and immunotherapy play important roles in HAL treatment and have gained clinical recognition. A bioinformatics analysis by Chen et al. revealed the EGFR, KRAS, and ALK pathways as potential targeted drug intervention routes for HAL (19). However, clinical HAL cases rarely show gene mutations, suggesting limited opportunities for targeted therapy or immunotherapy (20). However, Basse et al. reported on a patient who achieved a partial response with durvalumab, indicating immunotherapy as a potential new option for HAL (21). Moreover, studies have attempted to add sorafenib to the treatment regimen based on AFP positivity, referring to hepatocellular carcinoma treatment protocols (22). In the present case, based on this patient’s age, overall health status, and stage of disease, we did not consider surgery to be a viable first option. The patient was eligible for concurrent chemoradiotherapy. Additionally, if genetic testing had revealed favorable targets and positive immune expression, she could have chosen a combination of chemoradiotherapy and targeted therapy or chemotherapy combined with both targeted therapy and immunotherapy.

HAL is highly invasive and prone to multiple metastases (23, 24), leading to a poorer prognosis than that of NSCLC patients. Our patient’s advanced disease stage, age, and inability to afford relevant tests and treatments, along with her high AFP levels, suggested that sorafenib might be beneficial; however, she only received one round of platinum-based chemotherapy. Unfortunately, due to the patient’s poor compliance, she did not undergo the standardized serological and radiological follow-up after treatment. We were only able to learn from her son that her symptoms of cough, expectoration, and fever did not significantly improve after treatment. Moreover, she developed a loss of appetite and passed away shortly after treatment. Since no autopsy was conducted, we could not determine whether the patient’s death was due to the progression of the disease or the occurrence of adverse and unanticipated events.

Above all, we found that the patient had symptoms for an extended period without medical intervention at the onset of the disease. AFP testing was not performed during the initial diagnosis, and only issues related to inflammation were discovered through the lung biopsy. By the time the final diagnosis was established, the optimal timing for surgery had been missed, and during the subsequent treatment, the patient did not undergo genetic testing and refused radiation due to financial constraints. A literature analysis suggests that the patient might have had better outcomes given access to immunotherapy or targeted drug therapy. Given the severity of her illness, the patient appeared to have anticipated her demise and held little hope of treatment efficacy. Consequently, she opted for a chemotherapy regimen that would alleviate her financial burden while minimizing treatment-related discomfort. Although we presented her with multiple diagnostic and treatment options, we respected her choices and provided enhanced palliative care to ensure her comfort and dignity. Additionally, due to the patient’s poor compliance and the lack of standardized follow-up, it remained unclear if her death was caused by the progression of the disease or the occurrence of adverse and unanticipated events.

## Conclusion

HAL is a rare and challenging lung cancer subtype. This case and review highlight the need to consider HAL in cases of lung consolidations with necrosis and elevated AFP, relying on pathology and immunohistochemistry for diagnosis. Early treatment involves surgical resection, while advanced cases reference other lung and hepatocellular carcinoma treatments, which lack standardization and often have poor outcomes. Future research should explore molecular mechanisms and optimize treatment strategies to improve the survival and quality of life of HAL patients.

## Data Availability

The original contributions presented in the study are included in the article/**Supplementary Material**. Further inquiries can be directed to the corresponding authors.
