# Supplementary material for: Primary hepatoid adenocarcinoma of the lung with extremely elevated serum AFP: a case report and literature review
Source: Front Oncol. 2024 Oct 3;14:1448219. doi: 10.3389/fonc.2024.1448219 (PMC11484443; doi:10.3389/fonc.2024.1448219)
Supplement: Supplementary file 1 [file DataSheet1.docx]

Supplement 1


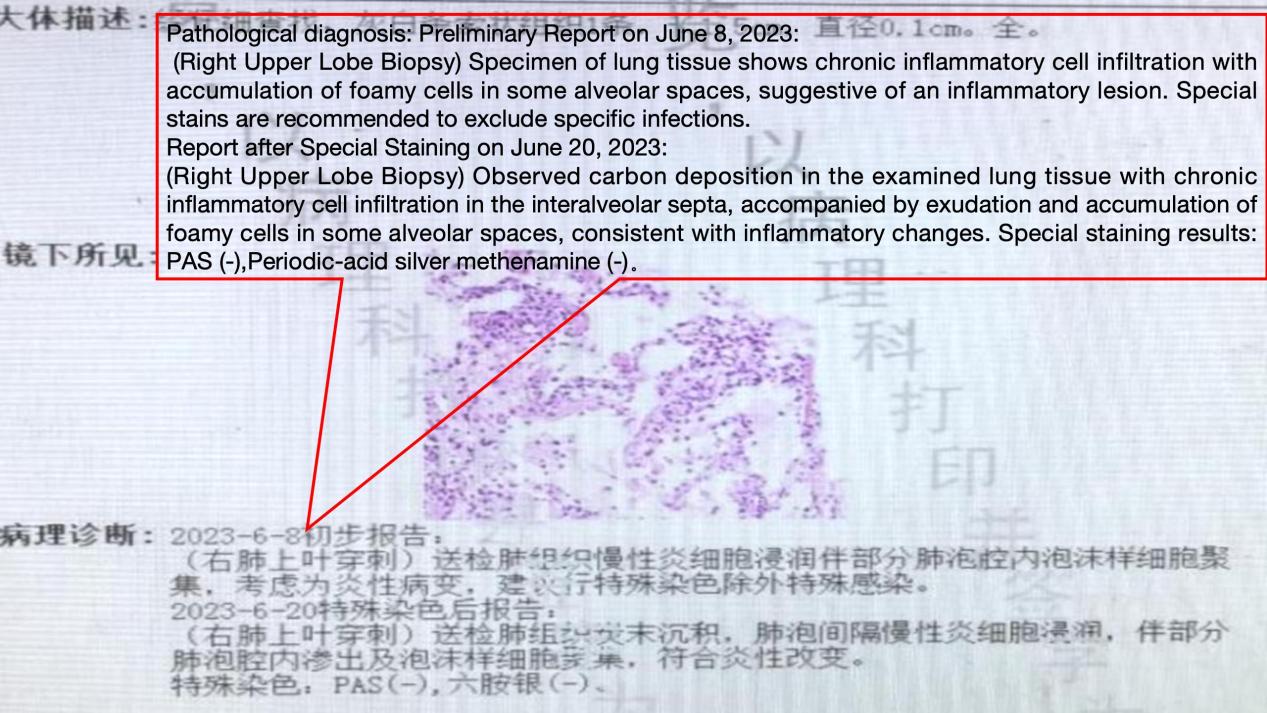


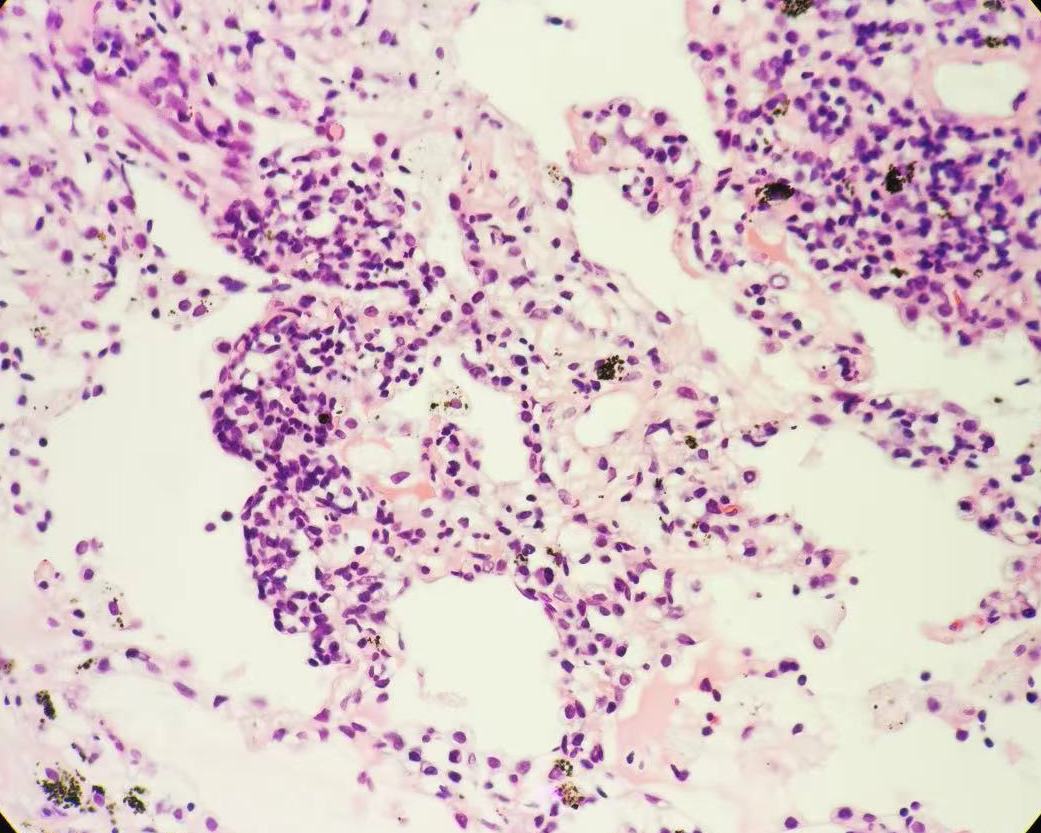


（The preliminary pathology report of the patient's lung biopsy indicated inflammatory changes. Further special staining was conducted to rule out infection, and the final report confirmed that the biopsy tissue was consistent with inflammatory changes.）

Supplement 2


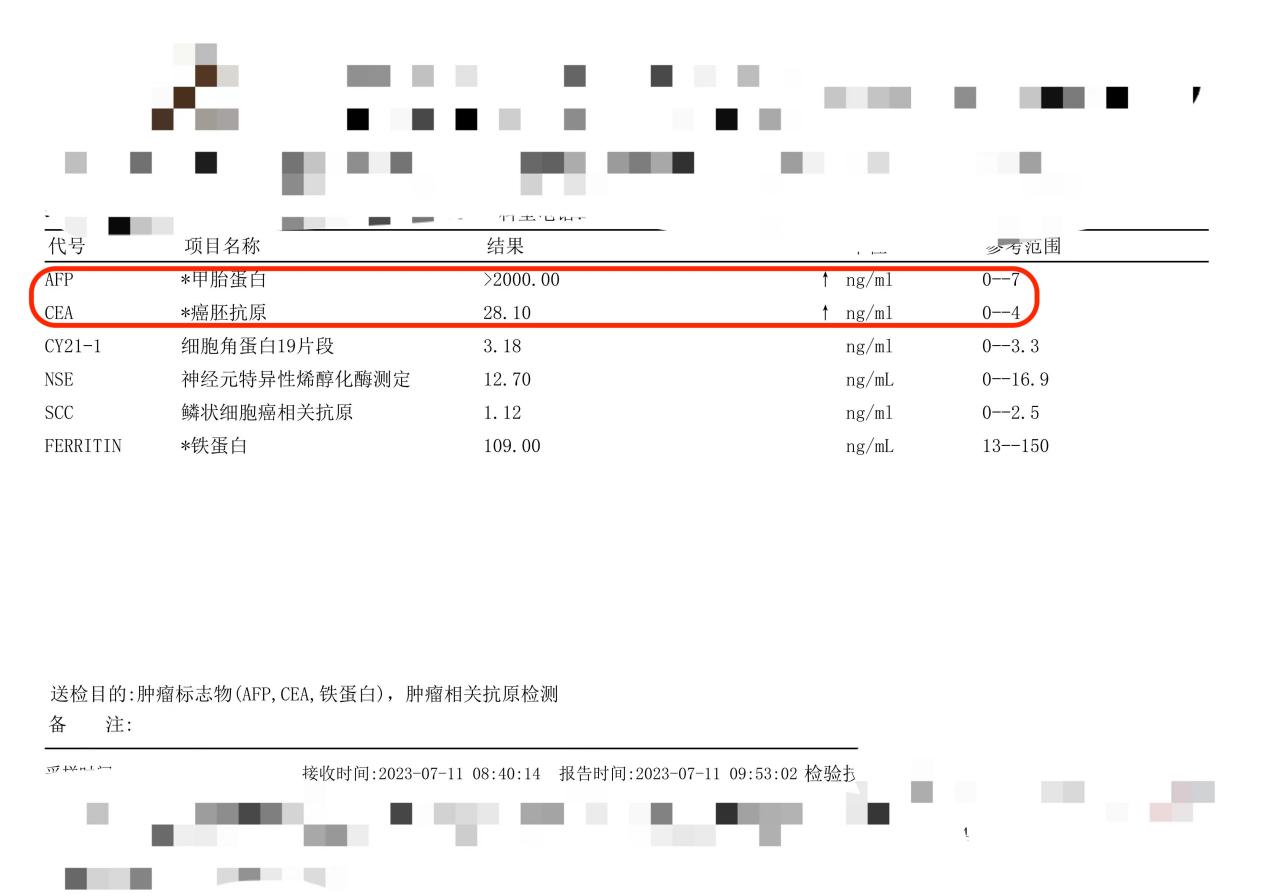


（The patient's serum AFP result is higher than 2000 ng/mL, and the CEA result is 28.1 ng/mL）

Supplement 3


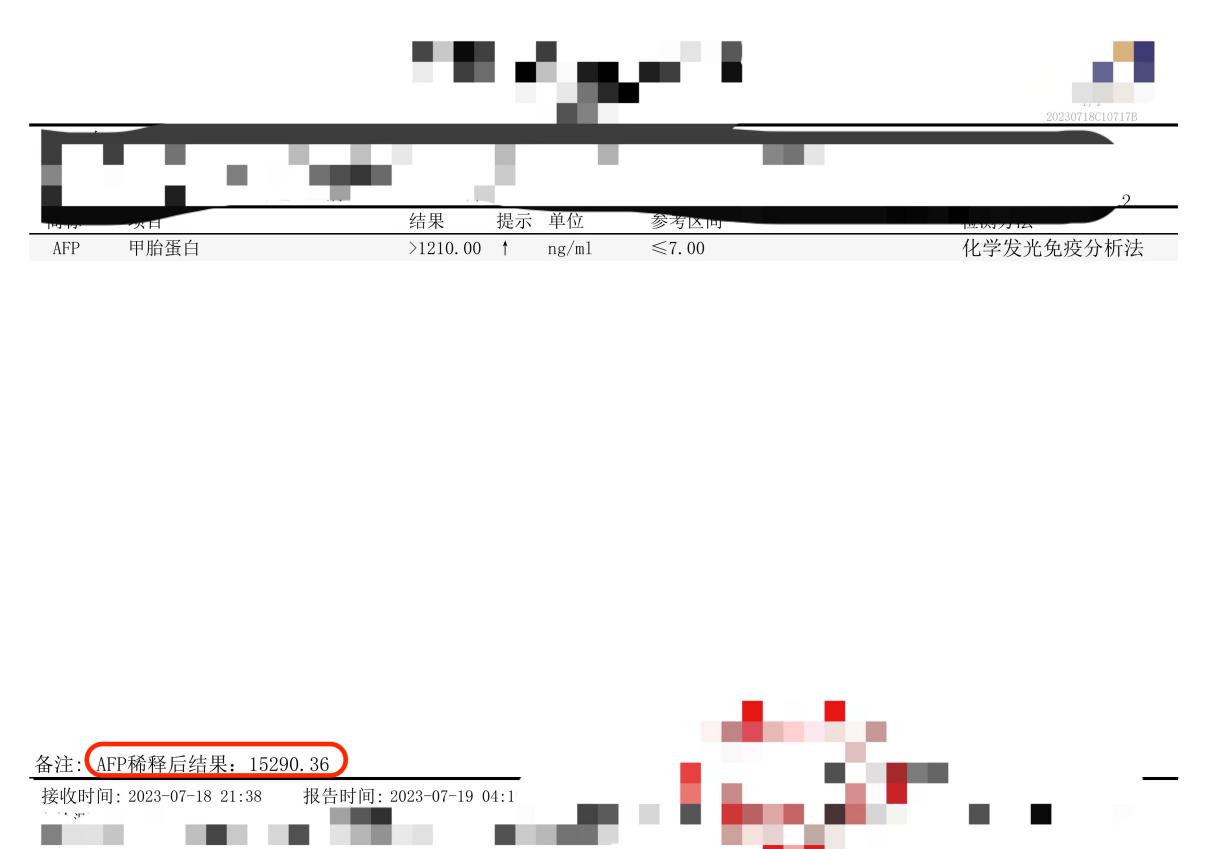


（Upon retesting the serum AFP using the dilution method, the final result was determined to be 15,290.36 ng/mL）
